# Diffusion in Colocation Contact Networks: The Impact of Nodal Spatiotemporal Dynamics

**DOI:** 10.1371/journal.pone.0152624

**Published:** 2016-08-08

**Authors:** Bryce Thomas, Raja Jurdak, Kun Zhao, Ian Atkinson

**Affiliations:** 1 Department of Information Technology, James Cook University, Townsville, Queensland, Australia; 2 Autonomous Systems, CSIRO, Brisbane, Queensland, Australia; 3 eResearch, James Cook University, Townsville, Queensland, Australia; Instituto de Fisica Interdisciplinar y Sistemas Complejos IFISC (CSIC-UIB), SPAIN

## Abstract

Temporal contact networks are studied to understand dynamic spreading phenomena such as communicable diseases or information dissemination. To establish how spatiotemporal dynamics of nodes impact spreading potential in colocation contact networks, we propose “inducement-shuffling” null models which break one or more correlations between times, locations and nodes. By reconfiguring the time and/or location of each node’s presence in the network, these models induce alternative sets of colocation events giving rise to contact networks with varying spreading potential. This enables second-order causal reasoning about how correlations in nodes’ spatiotemporal preferences not only lead to a given contact network but ultimately influence the network’s spreading potential. We find the correlation between nodes and times to be the greatest impediment to spreading, while the correlation between times and locations slightly catalyzes spreading. Under each of the presented null models we measure both the number of contacts and infection prevalence as a function of time, with the surprising finding that the two have no direct causality.

## Introduction

Complex networks [1–3] as a unified framework can describe a wide range of systems including the Internet and World Wide Web [4], biological systems [5–7] and social networks [8–10]. Dynamical processes taking place on networks, such as information diffusion or epidemic spreading, are strongly influenced by the structure and organization of networks [3]. For a long time, studies on the interplay between network structure and dynamical processes have been focused on static networks [11, 12]. Recently, attention has turned to the study of more complicated systems, in which dynamical processes are taking place on temporal networks with time-varying structures [13, 14]. Underlying factors in these systems that drive the evolution of temporal networks can subsequently impact the embedding dynamic phenomenon. Identifying these factors and their intrinsic association with the process is crucial for unravelling the complexity of these systems, as well as modelling, predicting and controlling the dynamic phenomenon.

Contact networks are one important instantiation of temporal networks that model either the logical or physical contacts between individuals. Examples of the former include long-range communications such as phone calls or email. Examples of the latter include colocation-driven short-range communications such as face-to-face human contact [15, 16] or proximity-based direct wireless transmissions [17]. Unlike logical contact networks, physical (i.e. colocation) contact networks are predicated on spatially constrained copresence and depend on mobility for broadscale spreading. The spatiotemporal dynamics of the actors (nodes) in such networks are therefore a critical determinant of spreading potential.

One of the best available sources of empirical data on colocation contact (also known as “encounter”) networks comes from electronic mobile wireless device traces [17]. These traces typically describe either directly recorded encounters between devices [18–21] or encounters which are inferred based on mutual presence at a known location [21, 22]. Much work has been devoted to the analysis of these traces, often in terms of their static network-theoretic properties and in some instances also in terms of spreading potential. For example, the authors in [20–22] all explicitly analyzed ad hoc [23] multi-hop message dissemination [24] facilitated by device mobility and encounters, the latter work being our own performed over the same trace analyzed in this paper.

One of the core tools for relating observed spreading behavior of a network quantity (e.g. virus, information) to idiosyncratic network features are *null models* [25, 26] which separately destroy correlations in a network’s contact structure. By simulating spreading on both the real and null networks one can establish to what extent certain correlations catalyze or impede spreading. Prior null models [25] have focused on *contact* shuffling—given a set of nodes connected by timestamped links, these models shuffle the links in one of a number of ways to retain certain correlations while destroying others. For example, the authors in [25] applied null model contact shufflings to a mobile call network (MCN) in which nodes were mobile subscribers and links were placed between caller/callee pairs and annotated with one or more call timestamps. A limitation of contact shuffled null models as applied to colocation-based contact networks is that they cannot draw any association between spatiotemporal dynamics and spreading. Rather, the models consider the contact network independent of the nature of the events which led to the contacts in the first place, such as the movement patterns of individual nodes in colocation contact networks.

While many attempts have been made to model such networks, there is yet no structured framework for establishing how nodes’ spatiotemporal preferences impede or catalyze spreading potential. This paper focuses on exploring the relationship between node spatiotemporal preferences and spreading potential in colocation contact networks. To address this question, we propose an alternative way of generating network null models through a method we refer to as *inducement* shuffling—shuffle the events on which contacts are themselves predicated and so in the process induce a modified set of contact events. Inducement shuffling possesses the attractive property of enabling second-order causal reasoning about spreading behavior. With contact shuffling it is only possible to state how characteristics of the contact network itself influence spreading. With inducement shuffling one can state how the nature of the events that lead to the contact network in the first place, such as the node movement dynamics, influence spreading. While both shuffling approaches lead to changes in network structure, inducement shuffling explicitly captures the relationships between the node preferences and correlations on one hand, and the spreading potential on the other.

We present our inducement-shuffled null models based on the theme of node, location and time which we use to decorrelate spatiotemporal node preferences. We apply the null models to an empirical trace in which (i) nodes are mobile wireless electronic devices such as smartphones, tablets and laptop computers (ii) locations are Access Points (APs) in a large university campus network and (iii) times are the times at which given devices were connected to given APs. Contacts in our empirical trace are predicated on device colocation. That is, two devices simultaneously present at a given location are connected by a timestamped link in the inferred contact network. Through inducement shuffling we destroy node’s time and location preferences, i.e. we decorrelate the relationships between nodes, times and locations. This in turn leads to a different set of colocation events and thus a modified contact network is induced. For the original and each induced contact network we simulate diffusion of a quantity starting from a randomly infected node under the Susceptible-Infected (SI) infection model [27]. To the best of the authors’ knowledge, the present work is the first to take a null models approach to isolating spreading impediments and catalysts in colocation-driven contact networks. More importantly, we believe the inducement-shuffled null models to be the first to enable reasoning about the second-order causal relationship between the events on which the contact network is predicated and subsequent spreading potential. Though motivated in the context of a wireless mobile device contact trace, we believe the inducement-shuffled null models presented in this paper may find broader applications in colocation contact networks, some well outside computer networks.

## Materials and Methods

### Dataset

Our work utilizes a wireless IEEE 802.11 (Wi-Fi) network trace collected by the University of Queensland (UQ) Australia describing device sessions at individual access points (APs) in a large university network over a 14 day period between Tue Nov 27 17:39:12 AEST 2012–Tue Dec 11 17:29:16 AEST 2012. Each session record in the UQ trace includes (i) the unique MAC address of the connecting mobile device (ii) session start time (iii) session end time (iv) AP name and (v) site. We clean the trace by discarding 1,462 sessions with no end time (active when trace collection ceased) and 1 session with zero duration (start = end). A further 1,279 sessions are discarded as specifying no AP. After cleaning, the UQ trace retains 546,260 sessions from 23,895 devices over 3,079 APs at a total of 24 discrete geographic sites. In this paper we focus only on the largest site in the UQ trace—the *St Lucia* campus. The St Lucia campus is a large university campus accounting for 445,867 sessions from 20,308 devices over 2,004 APs. After filtering on (v) to extract the St Lucia trace from the UQ trace, we then use (i)–(iv) to construct the minimum dataset for our analysis which consists of a set of session 4-tuples, each of the form 〈*N*, *T*_*start*_, *T*_*end*_, *L*〉. These 4-tuples fully describe which (*N*)ode (MAC address) partakes in a session at what (*T*)ime (start and end) and at what (*L*)ocation (AP), i.e. they encode the information about the spatiotemporal patterns of nodes. We refer to these 4-tuples simply as the “session tuples” from this point onwards. Note that nodes and locations just happen to be defined in terms of MAC addresses and APs respectively in our analysis. The notion of nodes and locations may be generalized to accommodate equivalent entities specific to the network under consideration.

### Contact Inference

The session tuples extracted from our original dataset and later reconfigured by our inducement shufflings do not explicitly describe contacts between pairs of wireless mobile devices. Rather, as is done in [21] we infer contact from colocation—two devices connected to the same AP at the same time are assumed to be in transmission range and so inferred as making “contact”. As noted by the authors in [21], this inference is an approximation. Devices connected to the same AP may not be able to communicate directly, devices connected to different APs may be able to communicate and some contacts take place outside the range of APs. Still, it is believed to be a reasonable approximation in the network under consideration. Contact inference translates the session tuples into a set of 5-tuples of the form 〈*N*_*i*_, *N*_*j*_, *T*_*start*_, *T*_*end*_, *L*〉, describing the location and duration of a contact between devices *N*_*i*_ and *N*_*j*_ (*i* ≠ *j*). For simplicity, and without loss of generality, we rely only on the first three fields of the 5-tuples in this paper i.e. 〈*N*_*i*_, *N*_*j*_, *T*_*start*_〉, which describe the initiation of contact events. We refer to these 3-tuples simply as the “contact tuples” from this point onwards. The contact duration is not considered here due to the lack of explicit session termination in the dataset, which creates a degree of uncertainty around the exact timing of session completion. Though this uncertainty is limited to the order of minutes, the incorporation of this uncertainty into the shuffling approach is out of scope of this paper and is left for future work.

### Contact Shuffling

As a preamble to our main inducement shuffling results, we apply the pre-existing contact-shuffled null models presented by the authors in “Small but slow world” [25] to our originally inferred contact network. This allows us to compare and contrast spreading behavior against that observed in prior work. We refer to these earlier null models simply as the “SBSW” or “contact-shuffled” null models. The SBSW models were previously applied to a number of contact networks, most notably a large Mobile Call Network (MCN) with timestamped links between caller/callee pairs. The authors use strings of capital letter abbreviations in naming the contact-shuffled null models, where each letter represents a retained correlation. These correlations are (D)aily pattern i.e. overall event frequency, (C)ommunity structure, (W)eight topology correlation (B)ursty event dynamics on single links and (E)vent-event correlations between links. Below we reproduce verbatim the description of the SBSW null models, more details about which can be found in the original paper:

- DCWB (*equal-weight link-sequence shuffled*): Whole single-link event sequences are randomly exchanged between links having the same number of events. Temporal correlations between links are destroyed.
- DCB (*link-sequence shuffled*): Whole single-link event sequences are randomly exchanged between randomly chosen links. Event-event and weight-topology correlations are destroyed.
- DCW (*time-shuffled*): Time stamps of the whole original event sequence are randomly reshuffled. Temporal correlations are destroyed.
- D (*configuration model*): The original aggregated network is rewired according to the configuration model, where the degree distribution of the nodes and connectedness are maintained but the topology is uncorrelated. Then, original single-link event sequences are randomly placed on the links, and time shuffling as above is performed. All correlations except seasonalities like the daily cycle are destroyed.

### Inducement Shuffling

Our main results are based around the inducement-shuffled null models presented below which are framed in terms of (T)imes, (L)ocations and (N)odes. The input to our inducement models are the session tuples. The output is a set of new session tuples of equal length with one or more of the correlations between pairs of T, L and N destroyed. We perform contact inference on the output sessions using the method just described to arrive at a set of new contact tuples. We reiterate that because inducement shuffling results in a different set of colocation events: it *implicitly* induces a modified contact network during contact inference, rather than explicitly reconfiguring the contact network itself.

The listing below describes each of the new inducement null models. Each model’s abbreviation is based on paired capital letters which indicate the retained correlation(s) between times, locations and nodes. For example, the null model LN-TN retains (L)ocation/(N)ode and (T)ime/(N)ode correlations. This means that, compared to the original traces, individual nodes still visit the same locations and they initiate sessions at the same start times. The change from the original trace is shuffling individual sessions relative to locations, thereby destroying the correlation between locations and times. Similarly, LN retains only (L)ocation/(N)ode correlation. Under this notation, the original session trace could also be referred to as LN-TN-TL as correlations are retained between all pairs of location, node and time. Note that our inducement models only destroy *correlations* between the three variables—they do not alter the independent frequency distribution of times, locations and nodes. In other words, nodes, locations and times with frequent activity maintain their activity levels in the shuffled trace. Moreover, session *durations* are always retained. That is, even if session times are shuffled, pairs of start and end times always move together. The inducement models are summarized in Table 1 in addition to the detailed listing below. Note that the final column of Table 1 includes spreading prevalence at 1 day, the meaning of which will become clear when we explain spreading dynamics shortly.

- LN-TN (*maintains the location visitation and session start times of nodes, while destroying correlations between time and location*): sessions are first grouped by node. For each grouping, the list of time pairs are shuffled (i.e. each start/end pair is exchanged with another pair in the same group). Grouping by node acts to “loosely bind” nodes and times—although the times are shuffled, they are always reallocated to the same node and therefore only the binding between times and locations is destroyed. i.e. LN and TN correlations are retained while TL correlation is destroyed.
- TL-LN *maintains the location visitation sequence of nodes and the correlation between time and location, while shuffling the node relation with session start time*: sessions are first grouped by location. For each grouping, the list of nodes are shuffled. Again, grouping acts as a loose binding mechanism keeping the same location/node association. i.e. TL and LN correlations are retained while TN correlation is destroyed.
- LN *maintains the location visitation sequence of nodes, while destroying the correlation between session times and both locations and nodes*: time pairs of the entire trace are shuffled. LN correlation is retained while TN and TL correlations are destroyed.
- TN *maintains the session times for nodes, while destroying the correlations between locations and both nodes and session times*: locations of the entire trace are shuffled. TN correlation is retained while LN and TL correlations are destroyed.
- TL *maintains the correlation between session times and locations, while destroying correlations between nodes and both session times and locations*: nodes of the entire trace are shuffled. TL correlation is retained while LN and TN correlations are destroyed.
- The empty set ∅ *destroys all pairwise correlations between time, location, and nodes, while maintaining the independent frequency distributions of all three variables*: locations of the entire trace are first shuffled and then nodes of the entire trace are shuffled. All correlations between time, location and node are destroyed.

**Table 1:** Null model summary and prevalence at 1 day ± standard error of the mean.

Shuffling	LN	TN	TL	|*I*(1 *day*)|/*N*
Original	✓	✓	✓	33.4%±0.6%
Group node, shuff. time	✓	✓		35.0%±0.6%
Group location, shuff. node	✓		✓	44.6%±0.8%
Shuffle time	✓			46.5%±0.8%
Shuffle location		✓		37.3%±0.6%
Shuffle node			✓	50.5%±0.8%
Shuffle location, shuffle node	-	-	-	50.3%±0.7%

In total there are 2^3^ = 8 potential models (including Original) based on which if any of the correlation pairs chosen from (LN, TN, TL) are retained during shuffling; however we omit one null model TL-TN from our analysis. TL-TN would in theory group by time and shuffle locations in order to destroy only the location/node (LN) correlation. Unlike location and node however, time is not a categorical variable and so grouping would require a discretization of times into an arbitrary number of slots. It is not readily apparent why one number of slots should be chosen over any other and so we leave contemplating TL-TN for future work.

We note that in our trace, locations are inherently discrete—individual APs with known identifiers. The inducement-shuffled null models do however generalize to broader settings, provided locations can be discretized. For example, a trace of mobile wireless bluetooth contacts annotated with known GPS coordinates might be discretized into a grid of lat/lon squares or geographically clustered. On the other hand, a contact network with no known locations (e.g. email) is not amenable to shuffling with the models presented in this paper. Moreover, though we focus our own simulations on a contact trace predicated on colocated wireless devices, the theme of times/locations/nodes is generalizable in the context of contact inference.

### Spreading Dynamics

We model spreading atop of all contact networks using the Susceptible-Infected (SI) infection model [27]. Nodes in the SI model are in one of two states: (S)usceptible or (I)nfected. State change is unidirectional with nodes graduating from S to I upon satisfying the infection condition—in our trace, colocation of a susceptible and infected device. Under the SI model, infection prevalence is monotonically nondecreasing as a function of time growing until all devices reachable from initial conditions are infected.

Similar to the work in [25], we start by first infecting a randomly chosen node at a randomly chosen contact event. The chosen event’s timestamp is interpreted as the simulation trial start time *t* = 0. We restrict the random sampling of the initial event to the first 4 days of trace to ensure a minimum of 10 days simulation “runway” before reaching the end of the trace. This 4/10 partition provides ample sampling opportunity across peak and trough traffic periods as well as weekdays and weekends while allowing enough simulation runway to observe the prevalence growth pattern. After sampling the initial infection event, we discard those contacts that occur either prior to the event or >10 days after the event. This ensures all simulations run exactly 10 days, preventing a non-uniform number of samples beyond the 10-day period (e.g. only initial infection events sampled from within the first 12 hours of trace would have known prevalence at 13.5 days). We proceed to calculate the prevalence denominator *N* as equal to the size of the Largest Connected Component (LCC) of the aggregated 10-day contact network which typically consists of the majority of all devices (>95%) in the period. Note that on rare occasions the initially sampled device will fall outside of the LCC. In this case we simply resample until the device is a part of the LCC. Starting at the initial infection and stepping chronologically through the contact sequence we then simulate the SI model of ideal diffusion whereby a susceptible device becomes infected upon contact with an already infected device. We denote the set of infected devices at time *t* as *I*(*t*) and the infection prevalence at time *t* as *P*(*t*) = |*I*(*t*)|/*N*. We perform a total of 250 random spreading trials (different starting nodes) and average the results. In the supporting material we also provide a comprehensive pairwise comparison of spreading potential for all inducement-shuffled null models along with standard errors around the averaged results.

## Results and Discussion

### Spreading Under Contact Shuffling

**Fig 1:**
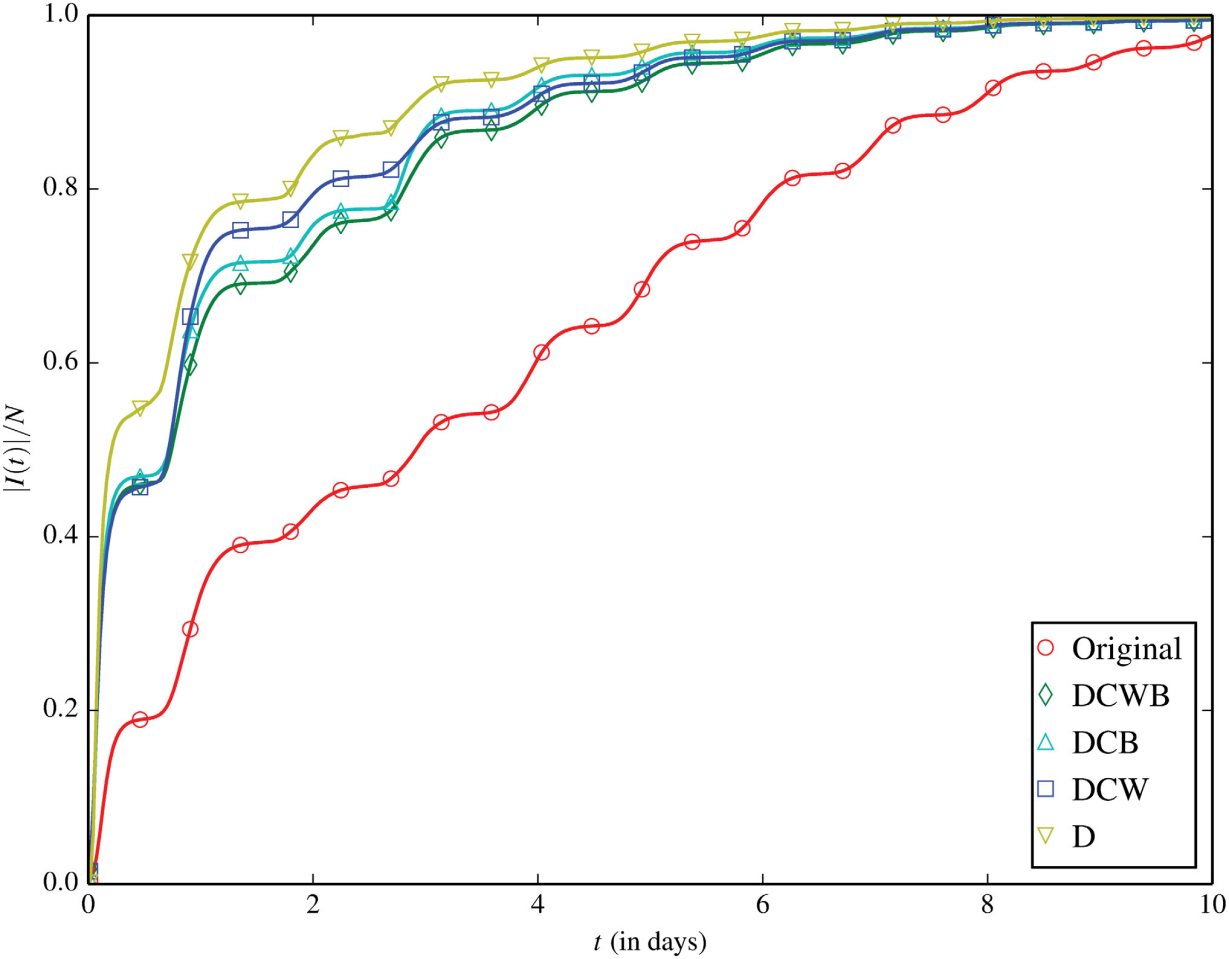
Fraction of infected devices |*I*(*t*)|/*N* as a function of time, for the original contact trace and “Small But Slow World” null model contact shufflings. Each line is based on a uniformly spaced sampling of prevalence over time. Note that the sampling resolution of lines is higher than their respective markers, as we only plot every *n*-th marker (*n* > 1) to minimize visual clutter. This (not fitting) is the reason for nonlinearity between plotted points. The same applies for the other plots presented throughout this paper.

In Fig 1 we present the spreading results under the pre-existing SBSW contact-shuffled null models on the St Lucia contact sequence. The pronounced “wavestep” pattern here captures the daily cycle of human activity well. We also note that the time to near full prevalence in our trace is on the order of a few days, remarkably shorter than the order (∼100 days) in MCN [25]. This is due to the fact that, in a Wi-Fi network successive transmissions can occur within a short period from one infected node to multiple susceptible nodes that simultaneously connect to the same AP, accelerating the whole spreading process.

Interestingly, our results show that the diffusion rate in DCWB is substantially faster than in Original, whereas DCWB and Original have been found to have a nearly identical diffusion rate in MCN [25]. This suggests that the temporal correlation between contact links is the most substantial impediment to the spreading in the Wi-Fi network. Further destroying link weights (DCB) or bursty single-link event sequences (DCW) only leads to a slightly faster diffusion. This also significantly differs from the observed effect of the similar null models in MCN, where the diffusion rates follow DCW > DCB > DCWB. We conjecture that link weights and bursty single-link event sequences make little difference in our trace due to the relative homogeneity of link weights—most device contacts occur only once (Table 2). This means destroying weight or bursty event correlations does little to perturb our contact network. By comparison, the MCN in [25] we calculate to have average link weight $\bar{\chi }\approx 34$ (306 × 10^6^ calls on only 9 × 10^6^ links), whereby destruction of link weights or bursty event sequences would lead to substantial perturbation. Finally, consistent with [25] we find D to spread fastest. We note however that the marginal difference is less than observed in the prior work.

**Table 2:** Number of repeat contacts between pairs of nodes.

repeats	count
1	385 453
2	70 092
> 2	96 298

### Spreading Under Inducement Shuffling

**Fig 2:**
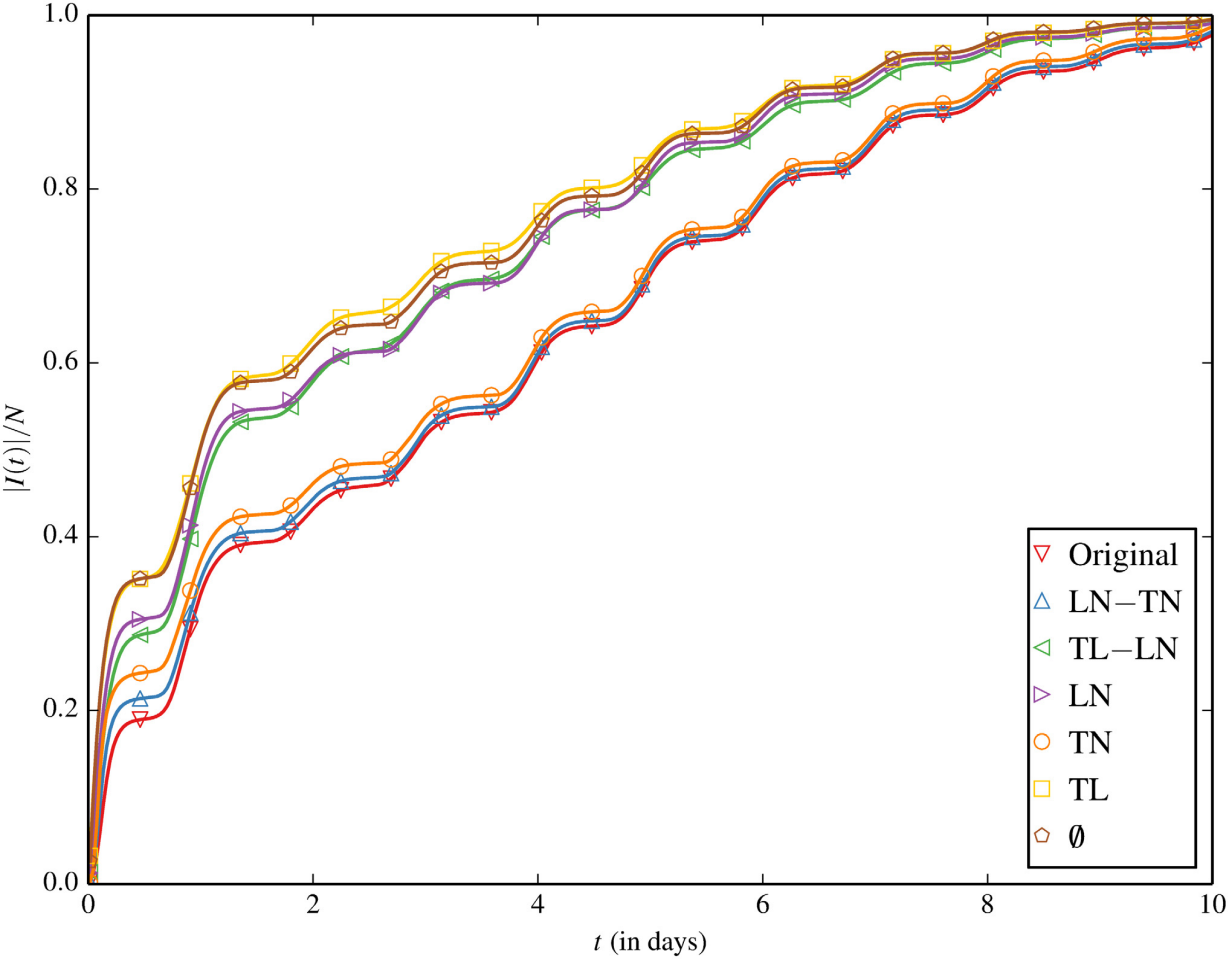
Fraction of infected devices |*I*(*t*)|/*N* as a function of time, for the original contact trace and inducement-shuffled null models.

**Fig 3:**
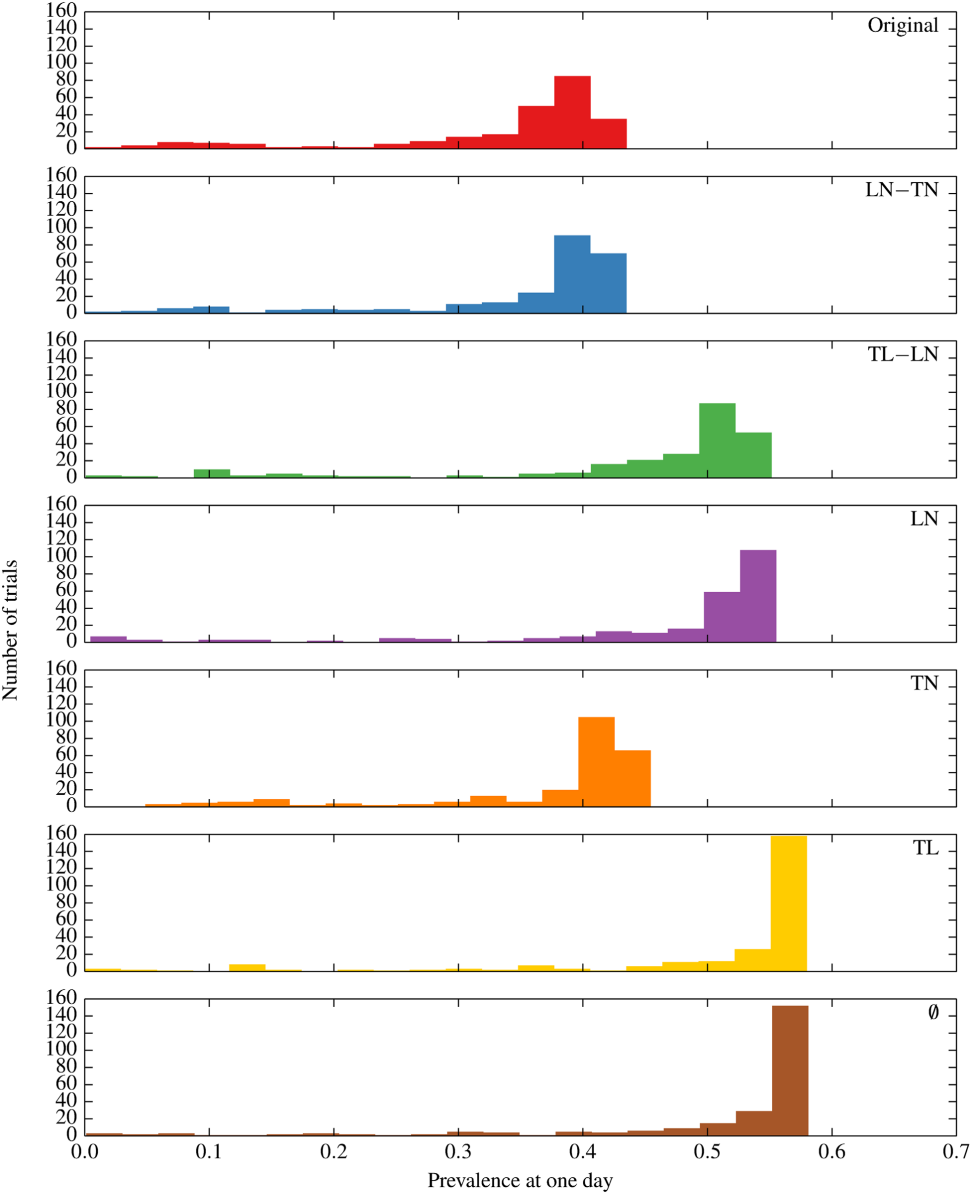
The histograms of the prevalence after one day under each null model.

We now present our main result in Fig 2—the subsequent rate of spreading as a function of time after inducement shuffling of the session records, which are averaged over 250 trials. The histograms of the prevalence after one day in different trials under each null model are shown in Fig 3 with the characteristics summarized in Table 1.

The original trace is found to spread slower than all of the inducement-shuffled null models, indicating the combined effects of location/node, time/node and time/location correlations result in the slowest spreading. In other words, the intrinsic coupling of movement patterns of the nodes in time and space seem to be impeding spreading by limiting randomness of contacts. Destroying the time/location correlation with LN-TN only marginally accelerates spreading and further destroying the location/node correlation in TN produces little further acceleration. This suggests that location-specific movement dynamics, which may attract nodes to some locations and repel them from other, are not the primary impediment to spreading. In contrast, destroying the time/node correlation with TL-LN substantially accelerates spreading. Maintaining the location/node correlations while destroying all temporal correlations (LN) also leads to relatively quick spreading, which provides further support that time/node correlations are responsible for slow spreading. Though it is interesting that some crossover is observed around 2–5 days between TL-LN and LN, there is little statistical strength to the statement that LN is ever truly faster which is made clear by comparing TL-LN and LN in S2 Fig (Supporting Information). TL and ∅ occupy the top echelon with respect to speed of spreading. This indicates two things: (i) location/node correlations have some impeding effect on spreading (TL being faster than TL-LN) and (ii) although TL produces more contacts than ∅ by keeping the correlation between time and location, both have a similarly fast spreading. The underlying reasons for this similarity will be explored in the next section. In summary, time/node correlation is found to be the main impediment to spreading with location/node correlation playing a smaller role.

### Contact Frequency and Spreading

To further explore the observed spreading phenomena, we study the contact frequency and its relation with the infection prevalence in each inducement-shuffling null model. In the spreading process under each null model, the infection prevalence at a specific time and the cumulative contact frequency upon that time are correlated, as they are both a monotonically increasing function of time. A question remains whether the infection prevalence is uniquely determined by the contact frequency across different null models. In other words, does infection prevalence and contact frequency have a direct causality. As we show in the following, there is no simple causality between these two, suggesting that the contact frequency is not sufficient to distinguish the spreading potential in different null models.

#### Contact Characterisation

**Fig 4:**
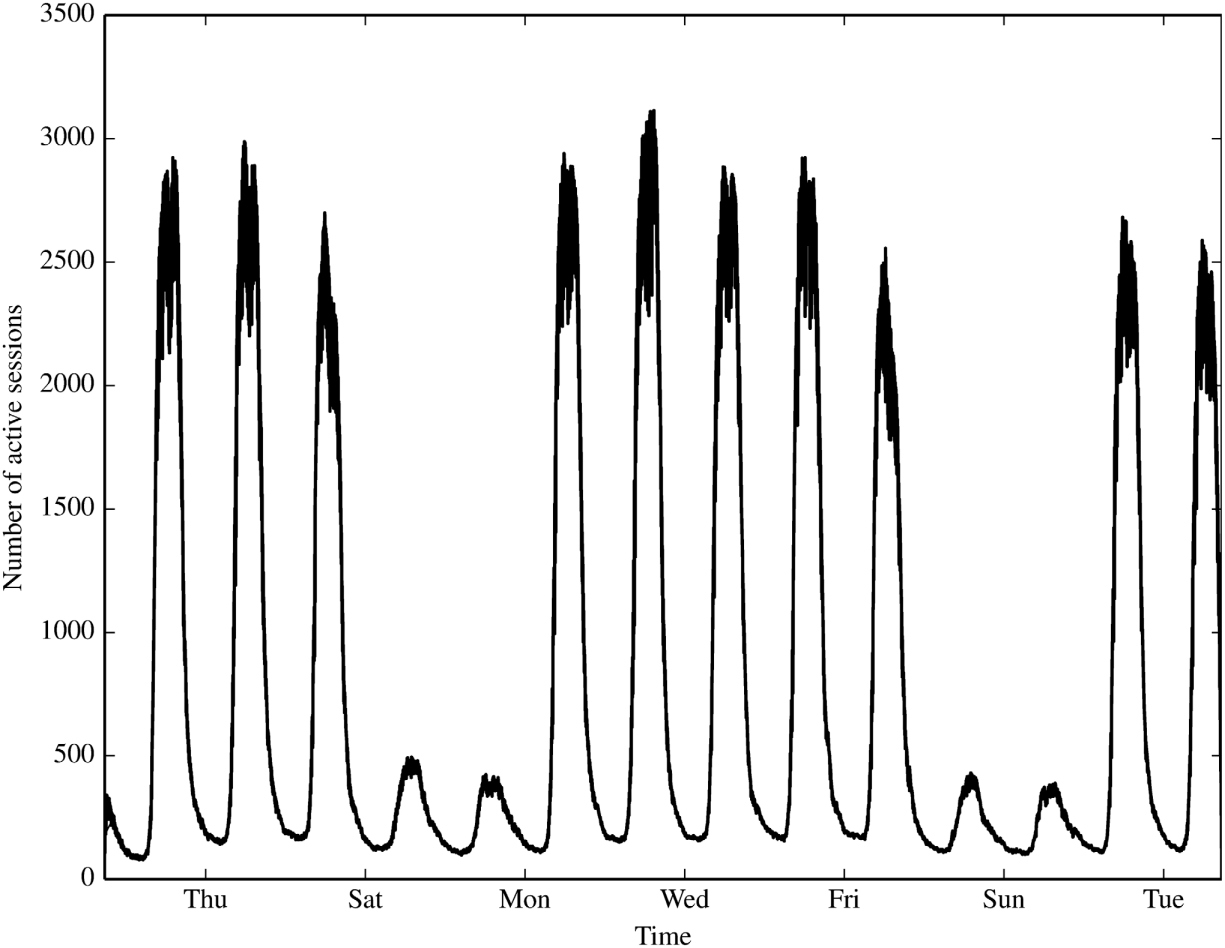
Session volume over time shows diurnal and weekday/weekend patterns.

**Fig 5:**
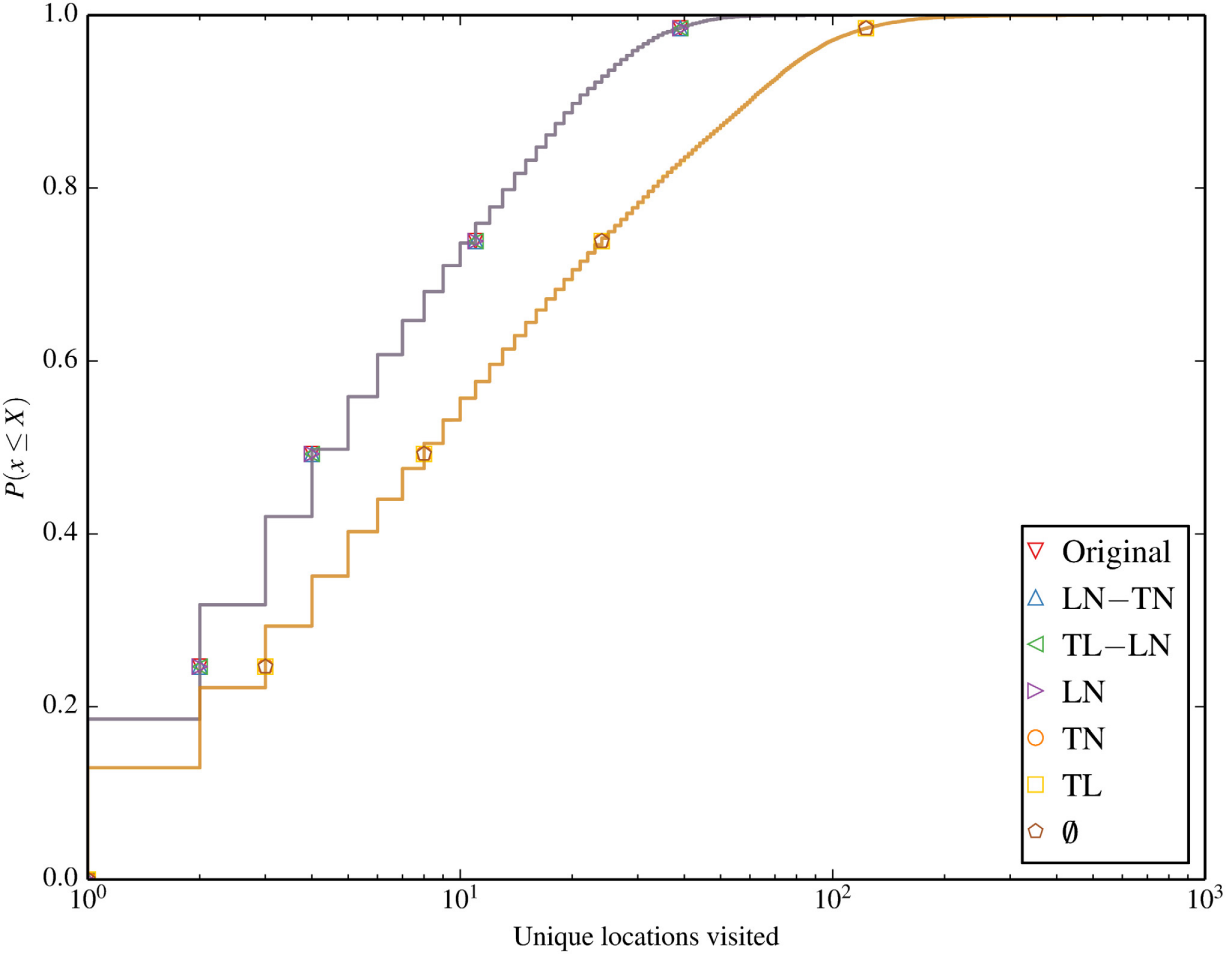
Unique locations per node. The number of locations per node show the increase of unique locations per node when LN is broken.

**Fig 6:**
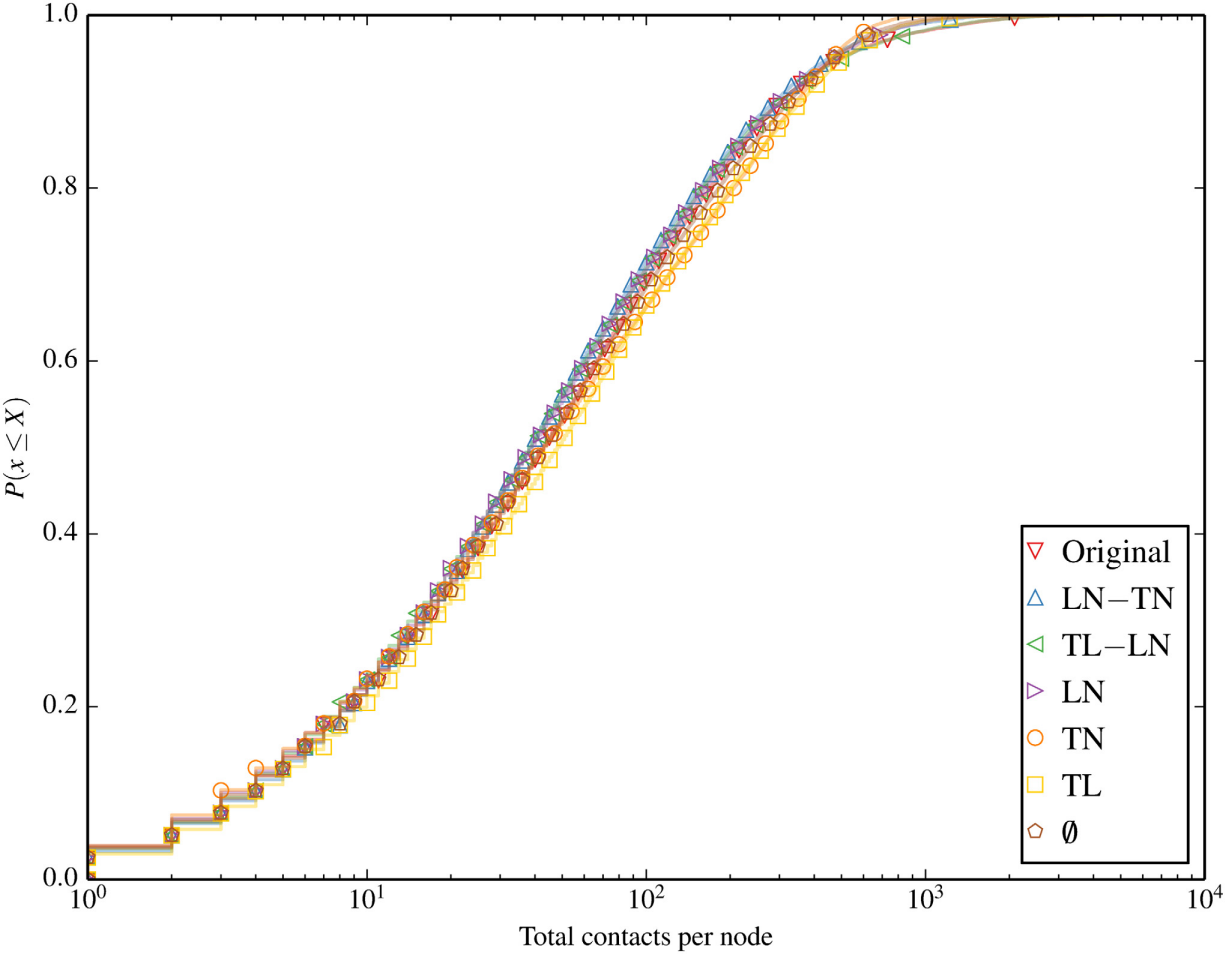
Total contacts per node. Total contacts per node are comparable across null models.

**Fig 7:**
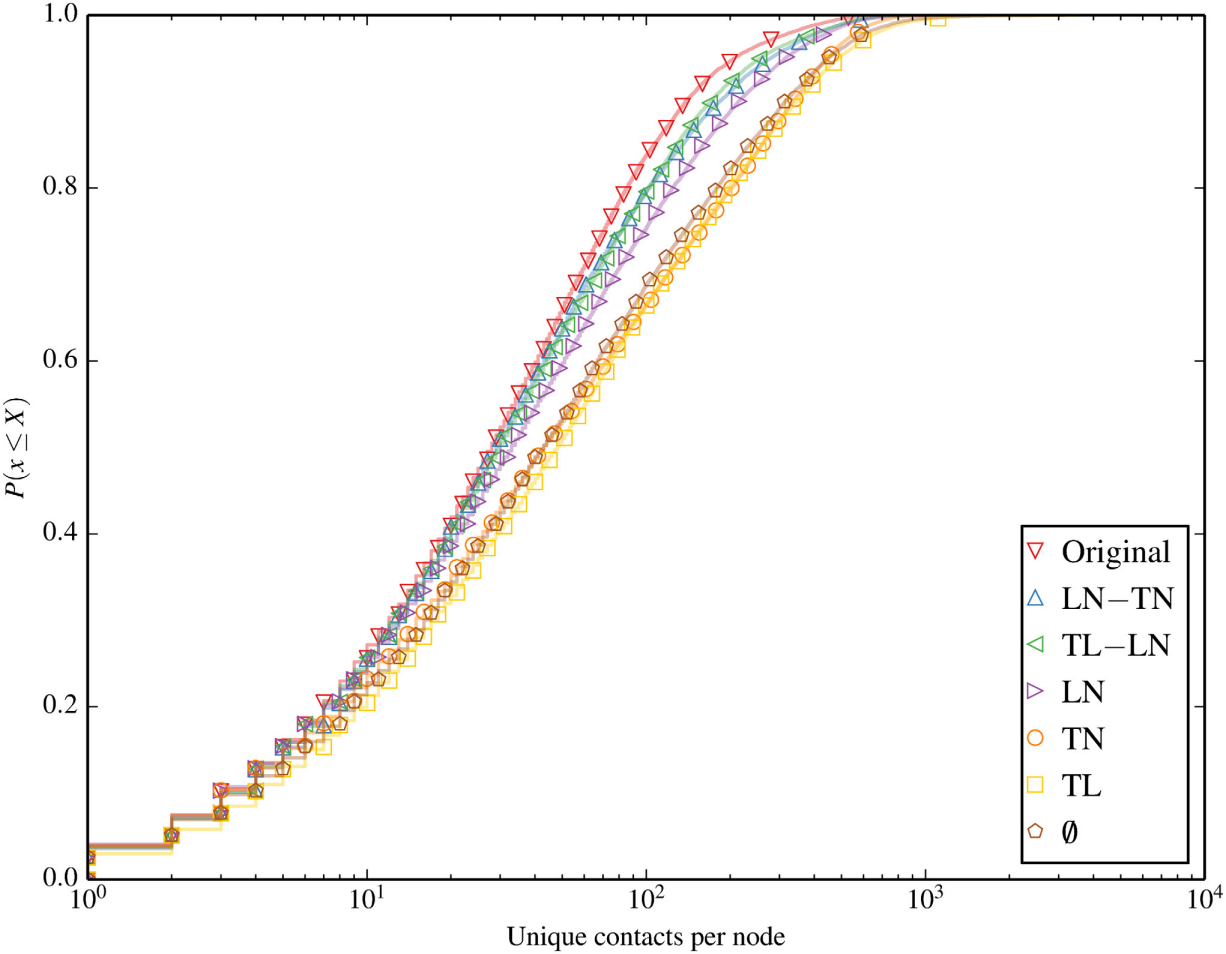
Unique contacts per node. Null models that destroy LN result in higher unique contacts.

We begin by characterising the contact and session statistics over time and space in Figs 4 through 7. Fig 4 shows the number of active sessions over time, clearly showing the differences in session volumes over weekdays and weekends and the diurnal patterns. We expect this to result in higher spreading propensity during weekdays when there are significantly more contacts. Fig 5 shows the ECDF of the unique locations visited per node for each of the inducement null models. A clear clustering of 2 sets of null models emerges, where all the models that destroy the location-node correlation (omit LN from their name) result in a higher number of unique locations visited by each node. Figs 6 and 7 compare the total and unique contacts per node, or put another way, the node degrees in the networks based on contacts. The total contacts distribution is comparable for all null models, with slight differences among the models. For unique contacts, we observe more pronounced differences between the models, where models that destroy LN (TL, TN, and ∅), produce the largest number of unique contact pairs. This effect is likely a result of the greater number of unique locations produced by these null models, which increases the likelihood of new unique contacts. LN produces the second highest number of unique contacts, while preserving any temporal correlation with LN results in fewer yet similar results. The original trace produces the least contacts, confirming that the inherent spatiotemporal node correlations limits unique contact opportunities.

**Fig 8:**
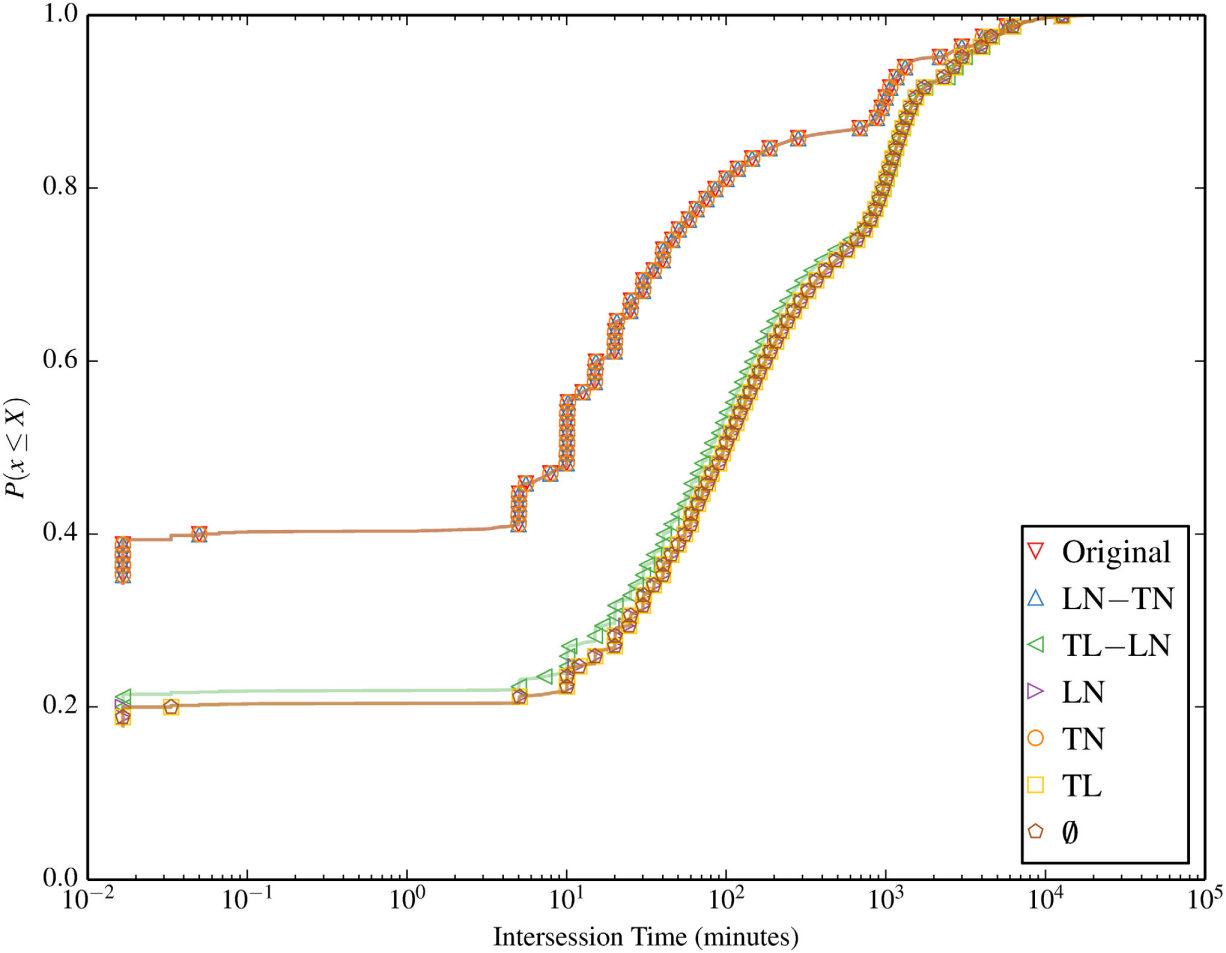
Intersession time. The intersession time distribution for each node is clearly longer for all null models that break TN.

The null models also impact the intersession times for each node, as shown in Fig 8. A clear separation of two groups of null models emerges between null models that break the node relation with session start time (omit TN), and those that preserve it. All null models that preserve TN have shorter intersession time, as the the temporal correlation between successive sessions is apparent. Large spikes in the plot account for typical session renewal times, such as at around 1 second when multiple attempts to establish a session occur, or at 5, 10, 15, and 20, 25, and 30 minutes, where access points typically update session status every 5 minutes. For null models that break TN, intersession times are significantly longer, emphasising the contribution of the time-node correlation to shorter intersession times.

#### Cumulative Contact Volume

**Fig 9:**
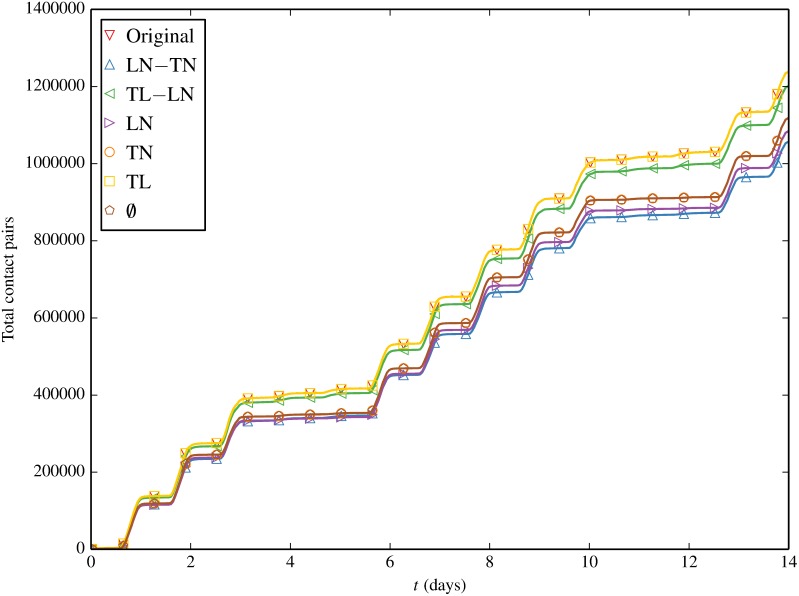
Total cumulative contacts as a function of time, for the original contact trace and contact traces induced by inducement shuffling.

We turn our attention next to elucidating the total contact counts in Fig 9. This effectively captures the total number of links in the network over time based on observed contacts, in contrast with the degree distribution shown above that illustrated comparable degree distributions for all null models. All contact tallys exhibit the characteristic diurnal stepping pattern of five weekdays interleaved with two weekend days. Original and TL grow at essentially the same rate (overlapping lines) as they both retain the correlation between times and locations leading to the same number of total colocation events. We say “essentially” rather than “exactly”, as all null models which decorrelate time and node (TL-LN, LN, TL, ∅) can occasionally produce “imaginary” contact events whereby a node is colocated with itself. Such events are discarded during contact inference and so are not accounted for in our tallys. For TL we find these imaginary contacts account for a negligible portion of all contact events (< 0.03%) and so TL appears to overlap Original. TL-LN on the other hand clearly illustrates the imaginary event phenomenon. In fact, the delta between Original and TL-LN in Fig 9 is a direct measure of the the number of discarded imaginary contacts under TL-LN shuffling which we find typically runs around 3%. It is interesting to note that LN is the only other shuffling with non-negligible imaginary events, again around 3%. The implication is that individual nodes likely have a strong affinity for specific locations (retained by *LN) leading to a (relatively) high number of imaginary events when the timing of node’s presence in the network is shuffled.

LN-TN produces the fewest total contacts indicating that correlated times and locations are the largest driver of contact volume in the Original trace. Further destroying either the location/node or time/node correlation as is done by TN and LN respectively increases total contacts. We expect that TN, LN and ∅ all produce the same number of total contacts as they all have the same expected number of nodes at a given location at a given time. The observed disparity of LN having fewer total contacts than TN and ∅ is simply attributable to the ≈3% of imaginary contacts produced and discarded under LN shuffling.

**Fig 10:**
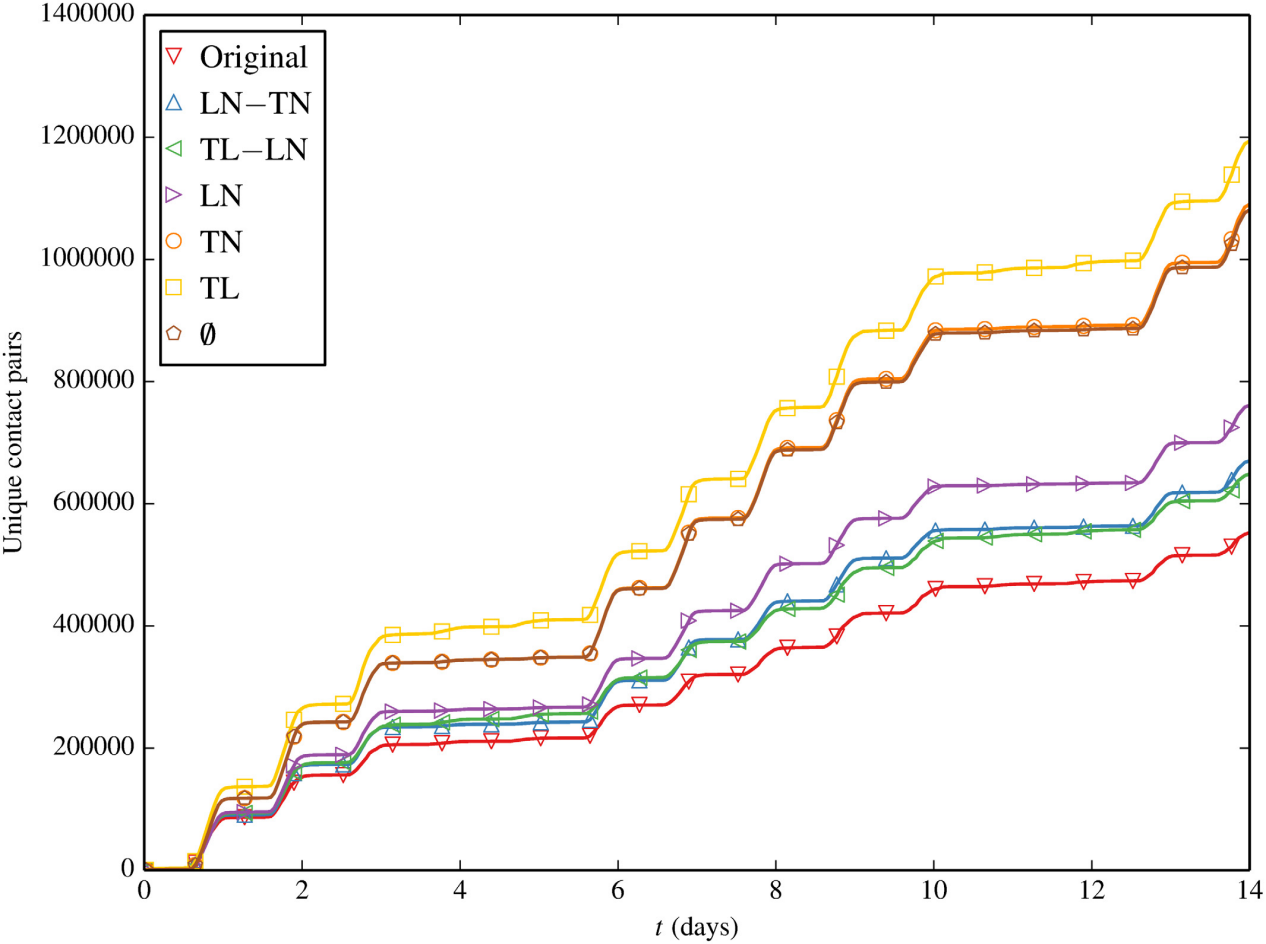
Unique cumulative contacts as a function of time, for the original contact trace and contact traces induced by inducement shuffling.

We now focus to the unique contact counts in Fig 10. Again, all contact tallys exhibit the characteristic diurnal stepping pattern being strongly driven by macro-scale periodic activity in the network. Whereas the original trace produces the most total contacts (in a tie with TL and TL-LN before discarding imaginary contacts), it also produces the *least* unique contacts. This suggests the “mixing” effect of the null models which increases unique contacts tends to be stronger than the opposite hindering effect the null models sometimes have on total contact volume. LN-TN and TL-LN produce approximately the same number of unique contacts which implies that destroying either time/location or time/node correlation has about equal effect on unique contacts. Destroying time/location and time/node correlations leaving only location/node correlation (LN) further increases unique contact. TN and ∅ produce still more unique contacts, possibly because the aforementioned affinity of devices for specific locations which is retained in LN had a stronger impeding effect on new unique contacts than does node’s time preferences. TL leads to the most unique contacts, likely because it retains the total contact boost driven by node’s preferences to be in the same location at the same time with the added benefit of mixing which nodes partake in the contact event.

As a final point of comparison between Figs 9 and 10 we note how unique contacts are always within a factor of 2.5 of total contacts. Again referring to Table 2, this is likely a by-product of a contact network whose original link “weights” as measured by repeat contact count are quite low versus the MCN analyzed in [25] (average link weight $\bar{\chi }\approx 34$).

Already Figs 9 and 10 appear to suggest that there is a weak relationship between a null model’s propensity to produce contact events and its subsequent spreading potential. We now proceed to plot contacts versus prevalence where this will become even more obvious.

#### Contacts vs. Prevalence

**Fig 11:**
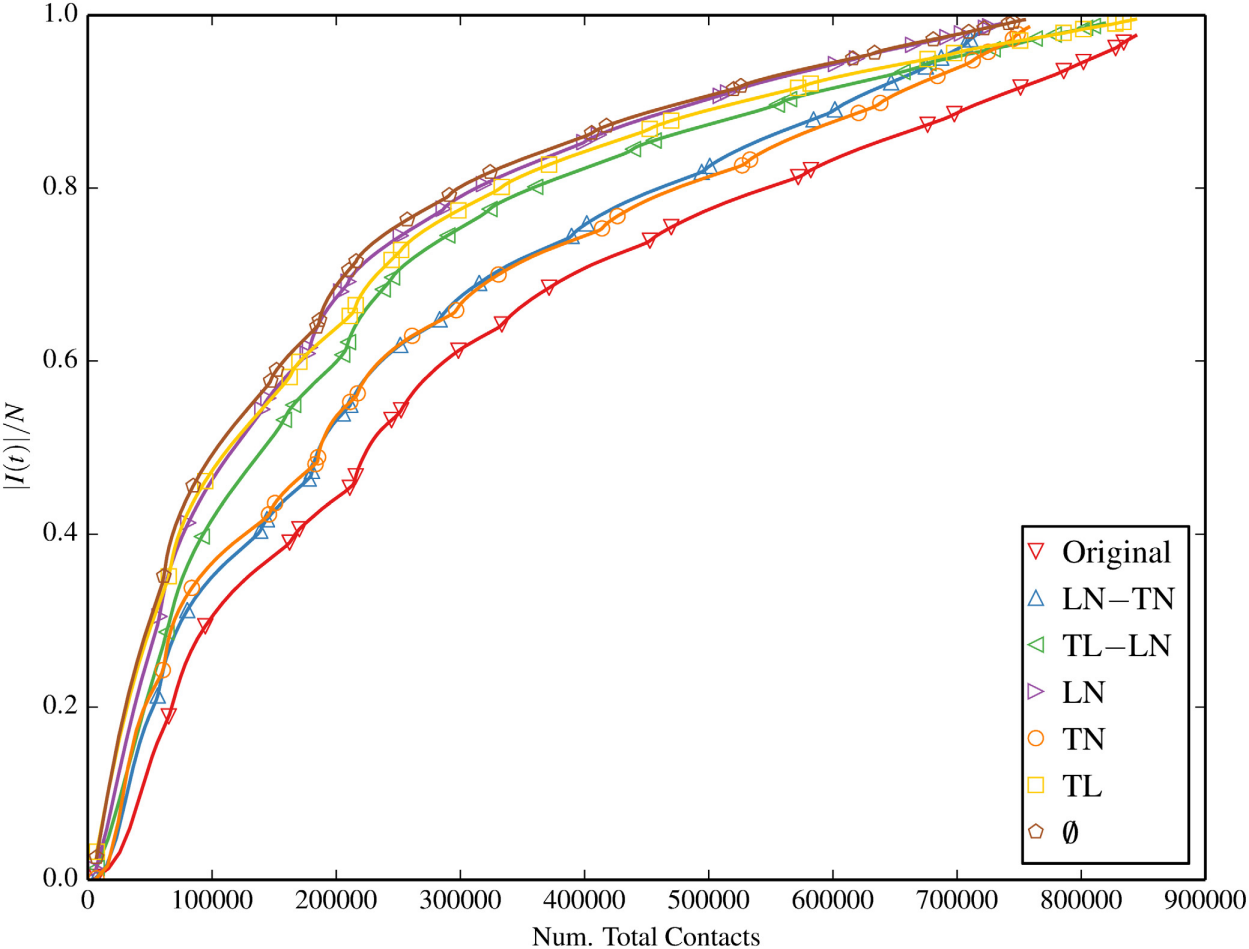
Fraction of infected devices |*I*(*t*)|/*N* as a function of the number of *total* contacts, for the original contact trace and inducement-shuffled null models.

**Fig 12:**
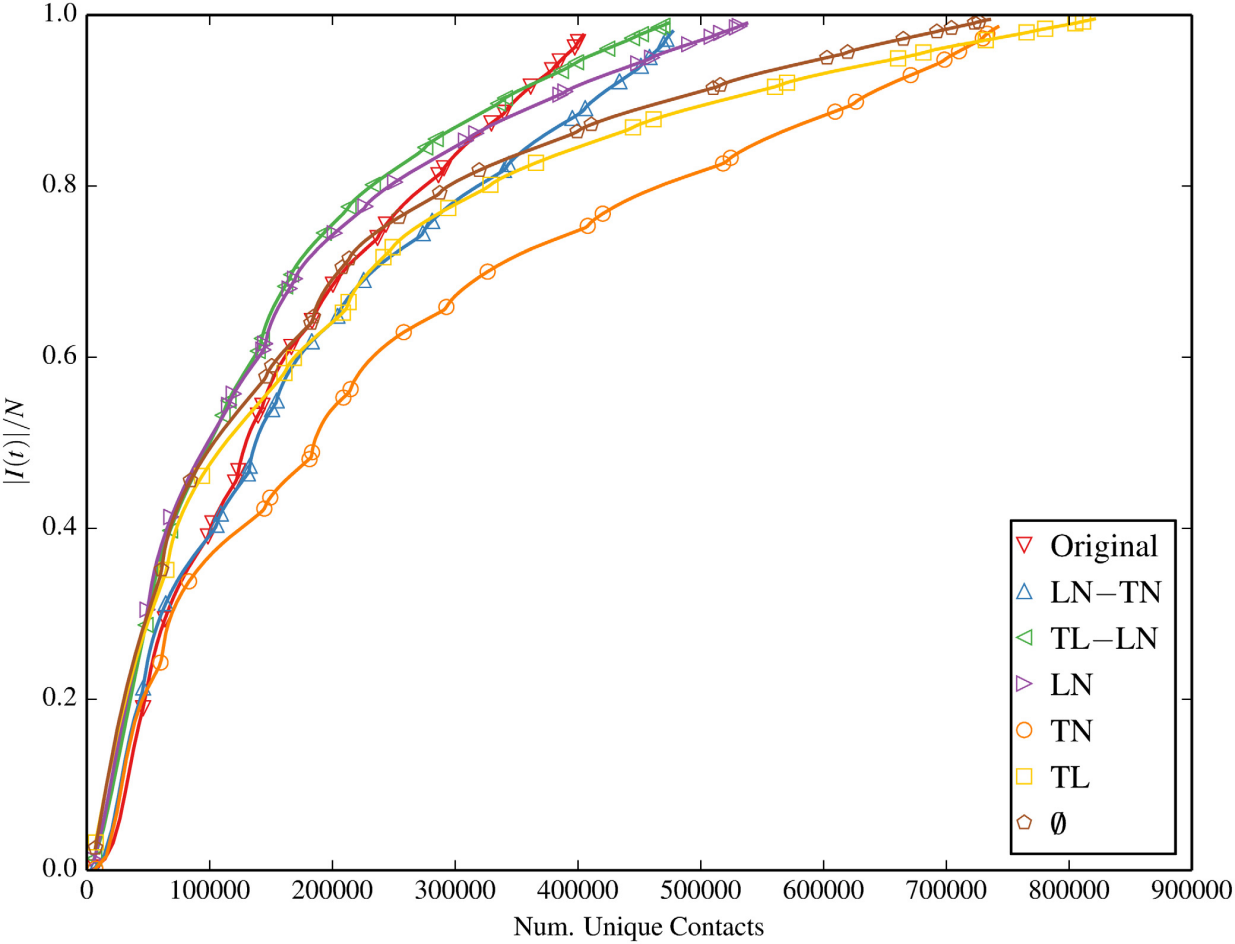
Fraction of infected devices |*I*(*t*)|/*N* as a function of the number of *unique* contacts, for the original contact trace and inducement-shuffled null models.

Figs 11 and 12 plot the infection prevalence as a function of the number of total and unique contacts respectively. It is evident that the relation between the infection prevalence and the contact frequency varies in different null models, suggesting that no direct causality exists. That is to say, the time of reaching a given prevalence in each null model is different, and the number of contact events upon this time is different as well. Therefore, without knowing the type of the null model, one is not able to infer the infection prevalence directly from the contact frequency. We can also interpret this phenomenon by using the term “spreading efficiency”, i.e. the number of contacts required to reach a specific prevalence. Our results demonstrate that the proposed null models have different spreading efficiency.

It is interesting to note that this disparity in spreading efficiency has also been implicitly observed both in this paper and prior work [25] under contact shuffling. This follows from the fact that contact shuffling inherently retains the same total contact frequency as a function of time, even after shuffling. Although unique (non-repeat) contacts may deviate from the original trace throughout the simulation period under D and DCW shufflings, these also equalize to that of the original trace by the end of the simulation. In any case, DCWB and DCB which match Original in both total and unique contacts at each time step present with drastically superior diffusion potential (on a per-contact basis) than the original trace in our analysis. We suggest that future work may wish to further explore the relationship between both the static and temporal structures of contact-shuffled and inducement-shuffled networks. Doing so may reveal common emergent properties such as statistical similiarities that offer a unified explanation to the disparity of spreading efficiency.

## Conclusion

This paper has analyzed impediments and catalysts to spreading in a contact network through the introduction of a set of inducement-shuffled null models which separately destroy the correlations between times, locations and nodes. The inducement-shuffled null models have enabled second-order causal reasoning about the observed spreading propensity. That is, how is spreading affected by the idiosyncratic behavior that lead to the observed contact network in the first place, rather than how the observed contact network alone affects spreading. Among our main observations is that (i) spreading is primarily impeded by time/node correlation and (ii) though correlations have a slowing effect in general, retaining time/location correlation alone proves an exception which slightly increases the rate of spreading versus a network with all correlations destroyed. Furthermore we have demonstrated a curious disparity between a null model’s ability to produce frequent contact events and its propensity to promote spreading. Finally, we have found that under pre-existing contact-shuffled null models temporal link correlations are the main spreading impediment in our trace, in contrast to earlier reported results.

In this study, we have assumed that co-location of two devices at an access point always leads to a contact between them, as in [21]. More specifically, our model assumes that the probability *p* of having a contact if two devices are co-located is always 1. Obviously, this assumption does not always hold, as there will be cases when devices are co-located at the same access point without being in contact, and the converse is true as well. An alternative is to consider a probability distribution for having a contact when two devices are co-located. The contact probability distribution can be either synthetic or empirical, and will be affected by spatial and temporal factors around the access point, such as the communication coverage area or users’ preferences for points of interest within the coverage area. Future trace-driven studies can collect explicit contact information among devices, where possible, for generating such a distribution. In fact, inferring contacts from co-location probabilistically is also broadly applicable to other mobility mining data sources, such as cellular phones [28] or geo-tagged social media traces [29], taking into account their respective positional uncertainty.

We conclude by proposing a number of avenues for future work. Firstly, there are several areas already alluded to in this manuscript. These are (i) defining and justifying appropriate grouped shuffling models on non-categorical variables such as time (ii) establishing if and how “imaginary” contact events between a node and itself ought to ever be avoided in shuffling and (iii) clearly articulating the conditions which may lead to large disparities between contact volume and prevalence. More broadly, it would be useful to explore whether the abstraction of times, locations and nodes generalizes well to other data traces of colocation contact networks, and indeed to other types of contact networks, particularly those which do not operate at human scale (e.g. cellular level networks). Complex networks in itself offers a unified framework for contemplating a diverse range of systems and so we expect that some subset of these may well be framed in a similar null model construction to that used in this paper. Moreover, there may exist alternative null model abstractions that will afford the same second-order causal reasoning about the nature of spreading in other types of contact networks. We urge the reader to consider what these abstractions might be.

## Supporting Information

S1 Fig Macroscale prevalences over time of all shuffling pairs compared. The x-axis of each subplot is time and spans the 10-day simulation interval, domain [0, 10]. The y-axis of each subplot is the column label’s prevalence subtracted from the row label’s prevalence and spans the range interval [-0.3,0.3] i.e. 30% either side of the blue dotted line which is centered at 0.0. The gray shaded region around each black line (very small in most plots but visible at high zoom) is the standard error of the mean of the row label’s prevalence. The gray shaded region around the center dotted blue line is the standard error of the mean of the column label’s prevalence.
(EPS)

S2 Fig Microscale prevalences over time of all shuffling pairs compared. This figure is the same as S1 Fig, the only difference being that the y-axis range has been zoomed to the interval [-0.015,0.015] i.e. 1.5% either side of the dotted blue line instead of 30% either side. This highlights the standard error of the mean more clearly and allows one to more easily establish regions of overlap between the reference and comparison’s standard errors giving a better idea of where differences are of lower statistical significance.
(EPS)
